# Investigation of the Maillard Reaction between Polysaccharides and Proteins from Longan Pulp and the Improvement in Activities

**DOI:** 10.3390/molecules22060938

**Published:** 2017-06-05

**Authors:** Miao-Miao Han, Yang Yi, Hong-Xun Wang, Fei Huang

**Affiliations:** 1 College of Food Science & Engineering, Wuhan Polytechnic University, Wuhan 430023, China; 18211673915@163.com (M.-M.H.); wanghongxun7736@163.com (H.-X.W.); 2 Sericultural & Agri-food Research Institute, Guangdong Academy of Agricultural Sciences, Key Laboratory of Functional Foods, Ministry of Agriculture, Guangdong Key Laboratory of Agricultural Products Processing, Guangzhou 510610, China; hf1311@163.com

**Keywords:** longan pulp, polysaccharide, protein, Maillard reaction, physicochemical property, activity

## Abstract

The purpose of this study was to investigate the Maillard reaction between polysaccharides and proteins from longan pulp and the effects of reaction on their in vitro activities. The polysaccharide-protein mixtures of fresh longan pulp (LPPMs) were co-prepared by an alkali extraction–acid precipitation method. They were then dry-heated under controlled conditions for monitoring the characterization of the Maillard reaction by the measurement of the free amino group content, ultraviolet-visible spectrum, Fourier transform infrared spectrum and molecular weight distribution. All the physicochemical analyses indicated the development of the Maillard reaction between polysaccharides and proteins. The in vitro activity evaluation indicated that the Maillard reaction could effectively enhance the antioxidant, antitumor and immunostimulating activities of LPPMs. The enhancement of 1,1-diphenyl-2-picrylhydrazyl radical scavenging activity and ferric reducing antioxidant power displayed both a positive correlation with the reaction time (*p* < 0.05). LPPMs dry-heated for three days obtained relatively strong inhibitory activity against HepG2 cells and SGC7901 cells, as well as strong immunostimulating effects on the nitric oxide production and tumor necrosis factor α secretion of macrophages. Maillard-type intermacromolecular interaction is suggested to be an effective and controllable method for improving the functional activities of polysaccharides and proteins from longan pulp.

## 1. Introduction

Longan (*Dimocarpus longan* Lour.), a subtropical evergreen tree belonging to Sapindaceae family, is commercially planted in China, Thailand, India and Vietnam [1]. Its mature fruits attract great attention due to its desirable flavor and abundant nutrients, and show an increase in production inrecent decades [2]. Several preservation methods, such as canning, freezing and drying, have been implemented to add value to this seasonal fruit because of its highly perishable nature, short storage life and susceptibility to postharvest diseases. Drying is the most common method for the industry to process fresh longan fruits [3]. Dried longan pulp is a kind of edible and pharmaceutical product, which has been widely used as a traditional Chinese medicine for its many health benefits [2,4]. Polysaccharides and polysaccharide-protein complexes have been confirmed to be the active macromolecules contributing to its antioxidant, antitumor and immunomodulatory activities [2,5,6,7,8], but those from fresh pulp have been rarely reported.

To understand the possible effect of the drying process on the bioactive polysaccharides of longan pulp, the physicochemical properties and immunomodulatory activities of polysaccharides from fresh pulp (LPF) and dried pulp (LPD) were comparatively investigated [9]. Compared with LPF, LPD contained more binding proteins via a stronger interaction force, and showed better immunostimulating effects on lymphocytes and macrophages. Meanwhile, the binding proteins were important to their activities, as the protein dissociation was associated with the weakening of activity [9].The structural difference of LPD from LPF was speculated to be the formation of polysaccharide-protein conjugates during longan pulp drying, and the potential mechanism might be related to the Maillard reaction between polysaccharides and proteins [10].

Maillard reaction refers to a complex group of reactions, beginning with the covalent bond between the amine groups and carbonyl compounds. Several previous studies have confirmed the Maillard-type conjugation between polysaccharides and proteins under dry-heating or wet-heating conditions, according to the typical changes inthe free amino group content, ultraviolet-visible (UV-visible) spectrum (or 420 nm absorbance), Fourier transform infrared (FTIR) spectrum and molecular weight distribution [11,12,13,14,15,16,17]. Compared with pure protein or protein-polysaccharide mixture, the conjugates formed by Maillard reaction generally exhibit improved features, such as emulsifying properties, antioxidant activity, thermal stability and solubility [10,12,13,14,15,16]. In addition, the binding proteins were recognized as the important structural feature contributing to the antioxidant, antitumor and immunomodulatory activities of polysaccharides [18,19,20]. However, the Maillard reaction between polysaccharides and proteins from longan pulp and its effects on their functional properties are still unclear.

Considering the growing production of longan pulp-dried products and the increasing attention on their bioactive macromolecules, the objectives of the present work were the following: (1) to verify the Maillard reaction of polysaccharide-protein mixtures of longan pulp (LPPMs) under dry-heating; (2) to investigate the effects of the Maillard reaction on the antioxidant, antitumor and immunostimulating activities of LPPMs.

## 2. Results and Discussion

### 2.1. Composition of LPPMs

An alkali extraction–acid precipitation method was used for the co-preparation of polysaccharides and proteins from longan pulp (i.e., LPPMs). LPPMs were composed of 28.15% proteins and 65.23% polysaccharides. Electrostatic interaction might be the primary driving force for the association of proteins and polysaccharides in the extracting solution [21]. The interaction between positively charged proteins and negatively charged polysaccharides resulted in the formation of soluble complexes first, and then the aggregation into insoluble interpolymers. Based on the hypothesis of weak polysaccharide–protein interaction via electrostatic force, it can explain why the binding proteins of polysaccharides from fresh longan pulp were mostly dissociated by mild ultrasonic treatment (150 W, 15 min) [9].

### 2.2. Maillard-Type Characteristics of LPPMs during Dry-Heating

#### 2.2.1. Free Amino Group Content and Uronic Acid Content

**Figure 1:**
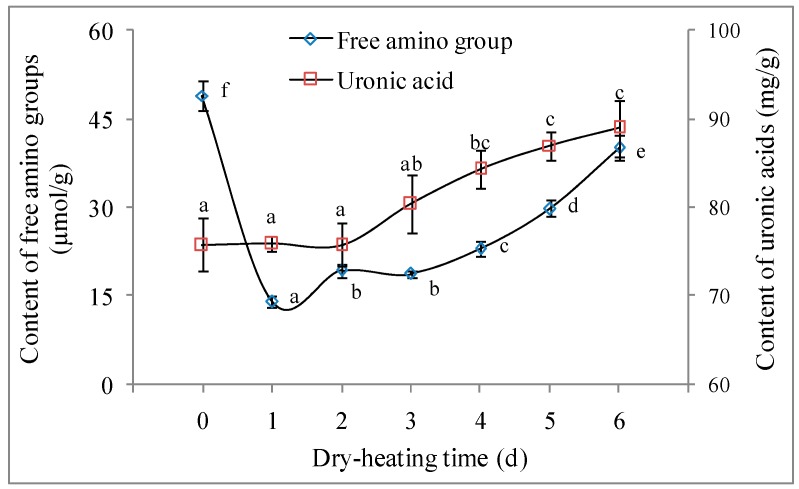
Change of free amino group content and uronic acid content of longan pulp polysaccharide-protein mixtures during dry-heating. The statistical difference (*p* < 0.05) among the samples dry-heated for 0–6 days is indicated by different letters. Data are represented as mean ± SD of three replicates.

The dry-heating method has been widely used for the preparation of polysaccharide–protein conjugate via the Maillard reaction [11,12,15,16,17]. The reaction between the carbonyl groups of polysaccharides and the free amino groups of proteins, together with the formation of a Schiff base, results in the decrease of free amino group content [13]. The free amino group content of LPPMs decreased from 48.91 μmol/g to 14.07 μmol/g after one day of dry-heating, but then began to increase (Figure 1). This change was similar as that of the Maillard reaction between canola protein isolate and gum arabic [14], implying the consumption and generation of free amino groups in the polysaccharide–protein reaction system. The loss of amines, due to the interpolymer formation through protein–protein interaction and protein–polysaccharide interaction [15,17], might be offset by the increased exposure of amine groups responding to the heat-induced unfolding of proteins [14]. In addition, the formation of a Schiff base between the carbonyl group and the amino group was followed by an irreversible Amadori rearrangement. The Amadori compounds might then undergo several degradation reactions, together with the generation of free amino groups and the cleavage of sugar units [22].

As an important factor associated with the bioactivity of polysaccharide [23,24], uronic acid content was investigated during the dry-heating of LPPMs. The uronic acid content did not change significantly in the first 3 days (*p* > 0.05), but then was increasing. The contents of LPPMs heated for 4–6 days showed no significant difference (*p* > 0.05), and the highest value of 88.96 mg/g was measured on the 6th day. In the Maillard reaction system composed of xylo-oligosaccharides and glycines (or prolines), uronic acid content increased as the reaction time prolonged, together with the decrease of reducing sugar content [25]. However, the potential mechanism related to the increase of uronic acid content in the polysaccharide–protein based Maillard reaction is unclear.

#### 2.2.2. UV-Visible Spectrum

**Figure 2:**
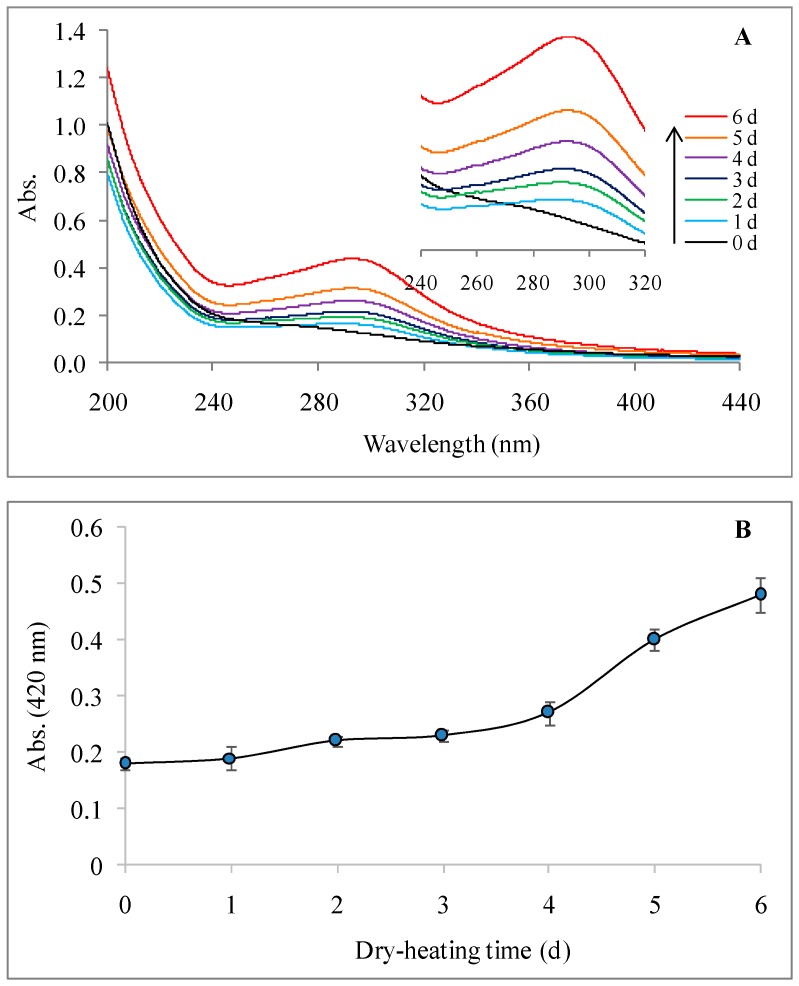
Change of ultraviolet-visible spectrum (**A**) and 420 nm absorbance (**B**) of longan pulp polysaccharide-protein mixtures during dry-heating.

As seen in Figure 2, the UV absorbance of LPPMs in the wavelength range of 280–320 nm obviously increased with increase of dry-heating time, as well as the visible absorbance at 420 nm. Heat can break the intramolecular forces which are responsible for maintaining the three-dimensional structure of a protein, thereby causing the protein molecule to unfold. The unfolding is typically accompanied by the exposure of some aromatic residues buried within the hydrophobic core, leading to absorbance increase in the wavelength range of 280–310 nm [26]. But it should not cause the increase of 420 nm absorbance (generally known as browning). Browning is an important feature of the advanced stages of the Maillard reaction, involving the colored products formed from aldol condensation and aldehyde–amine polymerization, together with the formation of heterocyclic nitrogen compounds [27,28]. Meanwhile, the increased absorbance at 294 nm can indicate the formation of uncolored compounds with near-ultraviolet absorption in the intermediate reaction stages [27,28]. It is suggested that the effect of dry-heating on the UV-visible absorbance of LPPMs can be ascribed to the Maillard reaction between polysaccharides and proteins. After dry-heating for 4 days, the reaction might enter the advanced stages.

#### 2.2.3. FTIR Spectrum

**Figure 3:**
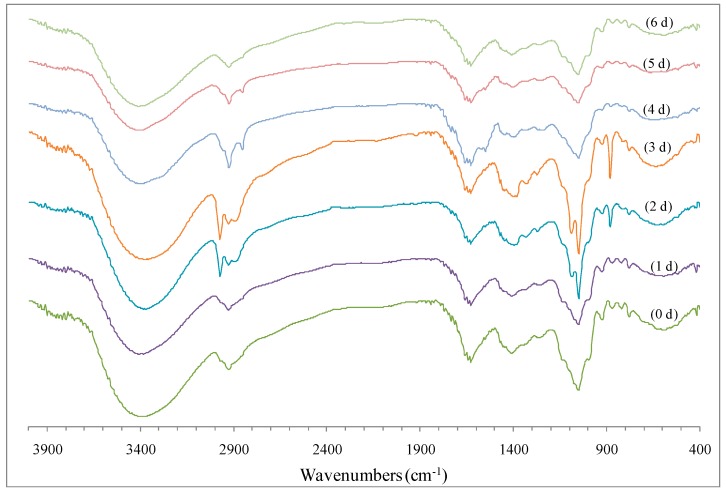
Change of Fourier transform infrared spectrum of longan pulp polysaccharide-protein mixtures during dry-heating.

FTIR spectroscopy is a frequently used as a technique for analyzing the structural features of polysaccharides and proteins. As shown in Figure 3, the characteristic absorption peaks of polysaccharide and protein were observed in the wavenumber ranges including 3380.96–3411.99 cm^−1^ (O–H and N–H stretching vibration), 2854.25–2928.16 cm^−1^ (C–H stretching vibration), 1628.23–1629.85 cm^−1^ (C=O stretching vibration and N-H bending vibration), 1383.53–1409.98 cm^−1^ (C–O stretching vibration and C=O symmetrical stretching vibration), 1049.85–1053.70 cm^−1^ (O–H bending vibration) and 924.31–924.78 cm^−1^ (the antisymmetrical ring vibration of d-glucopyranose), which were similar to the previous report [4].

FTIR spectroscopy can be also used to investigate the interaction between protein and carbohydrate [16,28,29]. The FTIR spectra of LPPMs dry-heated for different times showed obvious differences (Figure 3). The band changed in the amide I region (about 1650 cm^−1^), especially on the 4th day; this could be related to the generation of typical Maillard reaction products (i.e., a Schiff base imine group and enaminol group) and the formation of covalent bonds between carbonyl group and amino group [28]. The bands known as C–N stretching and N–H deformation in the amide II region (1480–1575 cm^−1^) and the amide III region (1240–1450 cm^−1^) displayed increasing intensities in the first 3 days, then gradually weakened. This change was similarly described in some previous studies [29,30]. In addition, other bands influenced by dry-heating were as following: the asymmetric and symmetric stretching vibration bands of alkyl groups (–CH_2_– and –CH_3_) at 2974 and 2854 cm^−1^; the N–H bending vibration band of amide II at 1551 cm^−1^; the C–O stretching vibration band of anhydroglucose ring at 1087 cm^−1^ [31]; the out-of-plane C–H bending vibration band of aromatic ring at 880 cm^−1^ [32]. All these results indicated the presence of the complicated Maillard reaction of LPPMs under dry-heating. Compared with polysaccharides from fresh longan pulp, those from dried longan pulp bound more proteins with stronger interaction force against ultrasonic dissociation [9]. It is suggested that Maillard-type conjugation between polysaccharides and proteins may also occur in longan pulp during hot air drying.

#### 2.2.4. High Performance Size Exclusion Chromatogram

**Figure 4:**
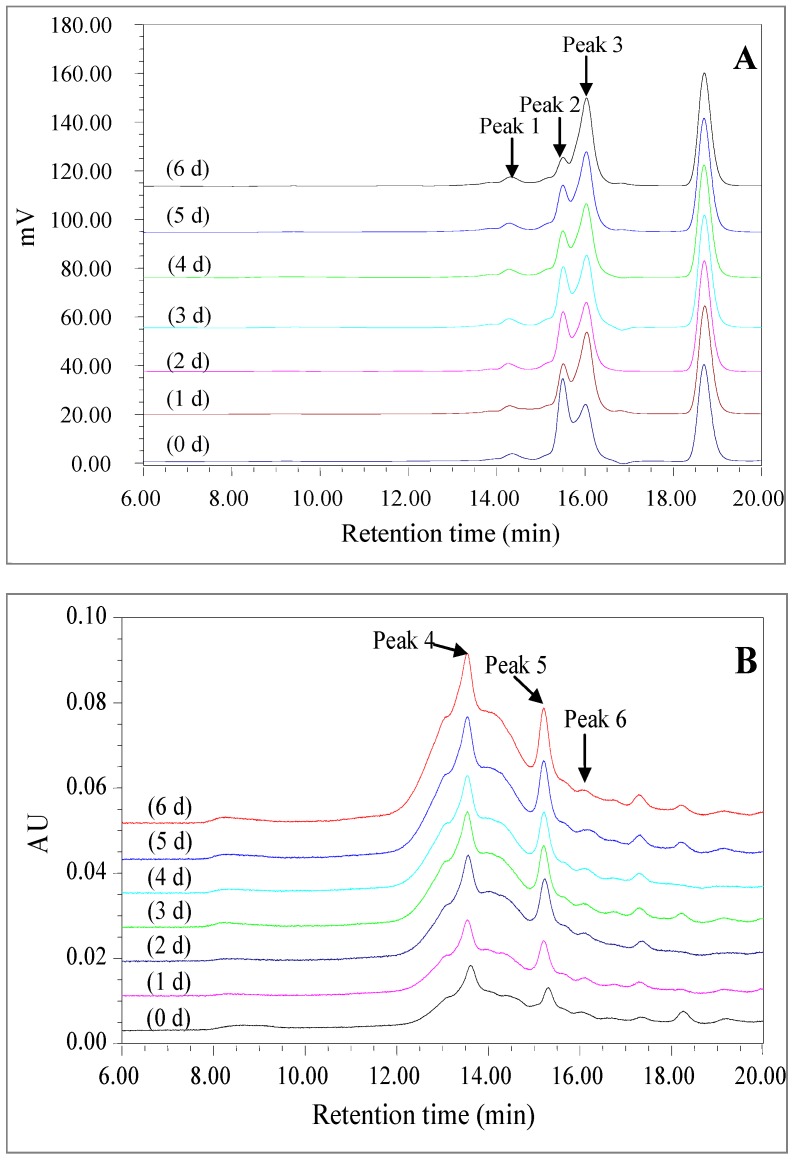
Change of molecular weight distribution of longan pulp polysaccharide-protein mixtures (LPPMs) during dry-heating. The high-performance size exclusion chromatogram of LPPMs was simultaneously performed with a refractive index detector (**A**) and a photo-diode array detector (**B**).

The effect of dry-heating on the molecular weight distribution of LPPMs was monitored using a high-performance size exclusion chromatography (HPSEC) method, as seen in Figure 4. Three peaks detected by the refractive index (RI) detector were the main fractions of LPPMs, which were numbered as peaks 1–3 (Figure 4A). The relative molecular weights of their peak values were in the ranges of 12.58–12.99 kDa, 8.95–8.98 kDa and 7.78–7.84 kDa, respectively. According to the chromatogram of blank control (not shown), the peak at retention time about 18.75 min belonged to the solvent. Although the main fractions showed no significant change in relative molecular weight during dry-heating, their peak area ratios obviously changed. Specially, the area ratio of peak 2 to peak 3 decreased from 1.06 to 0.43 in the first 2 days of dry-heating, increased to 0.67 on the 3th day, and then continually decreased to 0.25. The Maillard reaction between polysaccharides and proteins mostly produced conjugates with a higher molecular weight [11,12,16], and the conjugates might then undergo several degradation reactions after the Amadori rearrangement, resulting in the decrease of molecular weight [22]. The change of LPPMs in molecular weight distribution might be successively related to the elimination of electrostatic interaction between polysaccharides and proteins, the condensation between the carbonyl group and the free amino group, and the degradation of Maillard conjugates. There was a correspondence between the HPSEC-RI chromatogram and HPSEC-PDA chromatogram of LPPMs. The relative molecular weights corresponded to the peak values of peak 4–6 in the HPSEC-PDA chromatograms were in the ranges of 14.48–14.96 kDa, 8.68–8.94 kDa and 6.84–7.16 kDa, respectively (Figure 4B). But the differences among the protein signal-based chromatograms could not indicate the change of LPPMs in molecular weight distribution, because the absorption intensity of LPPMs at 280 nm was significantly enhanced as seen in Figure 2A.

### 2.3. Effects of Dry-Heating on the Activities of LPPMs

#### 2.3.1. Antioxidant Activity

As the main antioxidant mechanisms of polysaccharides have been indicated [33], their free-radical scavenging ability and reducing capacity were of major concern to investigate the effect of dry-heating on the antioxidant activity of LPPMs (Table 1). Compared with polysaccharides isolated from dried longan pulp by ultrasonic-assisted extraction [8], LPPMs showed stronger radical scavenging activities. The 1,1-diphenyl-2-picrylhydrazyl (DPPH) radical scavenging activity, hydroxyl radical scavenging activity and ferric reducing antioxidant power (FRAP) of LPPMs were all significantly enhanced by dry-heating for 1–6 days (*p* < 0.05). Especially, the DPPH radical scavenging activity and total antioxidant capacity gradually increased with increase of dry-heating time. Similar studies also indicated that polysaccharide-based (or protein-based) Maillard conjugates possessed stronger antioxidant activity than the unprocessed polysaccharide (or protein) [22,34].

Molecular weight and uronic acid content are considered to be the important factors affecting on the antioxidant activity of polysaccharide, although their relationships are still uncertain as many conflicting results reported [23,35,36,37]. They showed both significant correlations with the DPPH radical scavenging activity and the total antioxidant capacity of LPPMs (*p* < 0.05), but not the hydroxyl radical scavenging activity (*p* > 0.05). The free-radical scavenging mechanism of LPPMs might be principally due to the supply of hydrogen [38], and their reducing capacities might be associated with the presence of reductones which broke free radical chains by donating hydrogen atoms [39]. It was suggested that dry-heating might increase the number of active hydroxyl groups for the intramolecular hydrogen abstraction in LPPMs. The scavenging effects of LPPMs on the two free radicals were obviously different. Their hydroxyl radical scavenging abilities might be also related to the chelating to the metal ions which reacted with H_2_O_2_ to give hydroxyl radicals [40].

#### 2.3.2. Antitumor Activity

**Figure 5:**
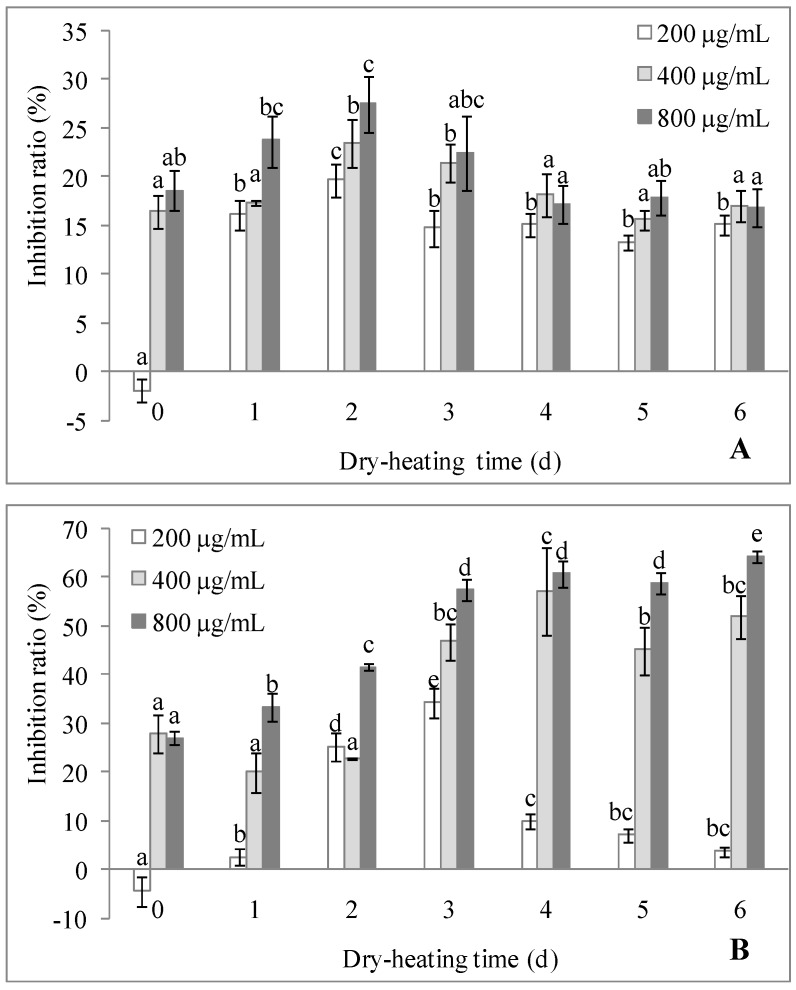
Antitumor activities of longan pulp polysaccharide-protein mixtures dry-heated for different time periods. The proliferative inhibition against HepG2 cells (**A**) and SGC7901 cells (**B**) were measured by the cell counting kit-8. The statistical difference (*p* < 0.05) among the samples at the same dose is indicated by different letters. Data are represented as mean ± standard deviation of six replicates.

The in vitro antitumor activity of polysaccharides from dried longan pulp were tested on HONE1 cells, A549 cells, HeLa cells and HepG2 cells, and the highest inhibition ratio (about 30%) was found at the dose of 200 μg/mL against HepG2 cells [5,6]. In addition to HepG2 cell, tumor cell SGC7901 was chose for the in vitro evaluation of antitumor activity of LPPMs. The inhibition ratios of heated and unheated LPPMs against the two cells showed no obvious correlation with dose in the range of 50–800 μg/mL, and the inhibitory effects were mostly obtained at high doses, as seen in Figure 5. The inhibitory effect of LPPMs against HepG2 cells was significantly enhanced by dry-heating for 2 days (*p* < 0.05), with the inhibition ratio of 27.46% at 800 μg/mL. The inhibition ratio then decreased with the increase of dry-heating time. At the same dose, the HepG2 cell inhibition ratios among samples heated for 4–6 days showed no significant difference (*p* > 0.05).

The inhibitory activity of LPPMs against SGC7901 cells could be also improved by dry-heating (Figure 5B). At the dose of 200 μg/mL, LPPMs did not inhibit the proliferation of SGC7901 cells initially, but obtained the inhibition ratio of 34.39% after 3 days of dry-heating. LPPMs heated for 3 days exhibited the inhibition ratio of 46.83% and 57.40% at 400 μg/mL and 800 μg/mL, respectively. The values showed no obvious difference from those of LPPMs heated for 4 days or 5 days (*p* > 0.05). The highest inhibition ratio of 64.37% belonged to 800 μg/mL of 6-day heated LPPMs.

After incubation with 800 μg/mL LPPMs for 12 h or 24 h, the number of live macrophages significantly increased (data not shown). The previous studies also indicated that polysaccharides and the polysaccharide–protein complex from longan pulp had no significant cytotoxicity against immune cells [4,7,9]. It was suggested that the cytotoxicity of LPPMs against non-tumor cells could be ignored. Protein-binding may be beneficial to improve the antitumor activity of the polysaccharide [20]. It might promote the interaction between LPPMs and negatively-charged tumor cell membranes to trigger antitumor events, by increasing the positive net charge in polysaccharide molecules. Polysaccharides with high molecular weights are generally believed to have stronger antitumor activity [41]. The uronic acid contents of *Pleurotus eryngii* polysaccharides exhibited a positive correlation with their antitumor activities against HepG2 cells [24]. However, the relationship between the structure and antitumor activity of LPPMs, involving in the complicated effect of the Maillard reaction, is unclear.

#### 2.3.3. Immunostimulating Activity

**Figure 6:**
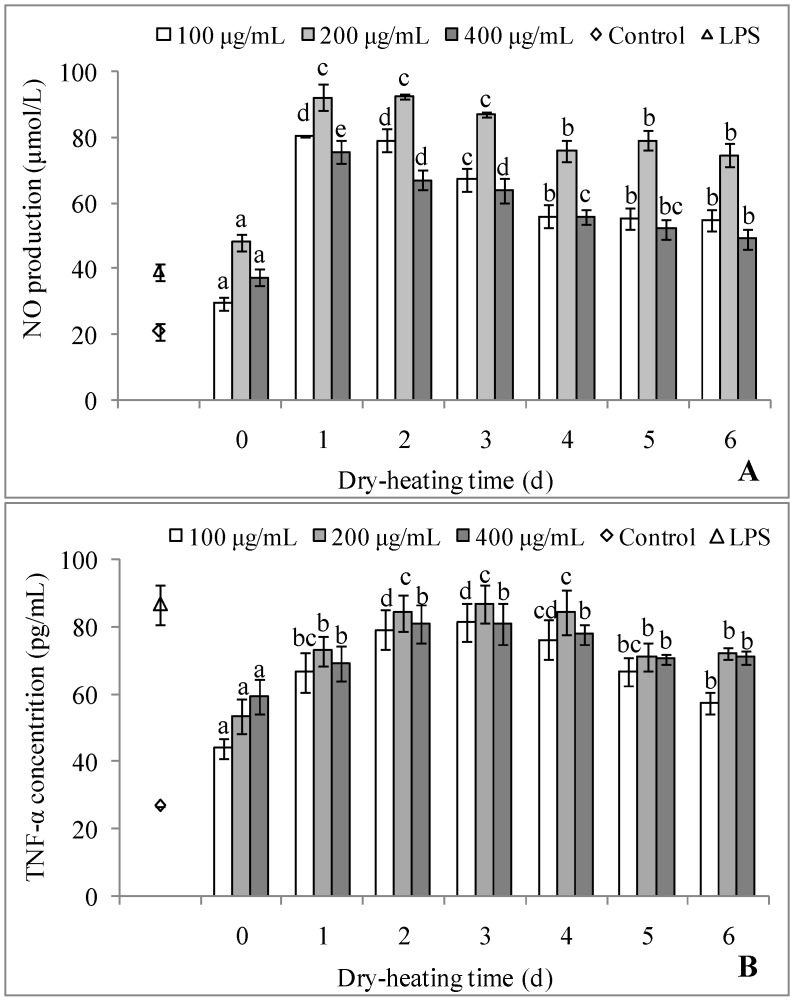
Macrophage-stimulating activities of longan pulp polysaccharide-protein mixtures dry-heated for different times. The nitric oxide production (**A**) and tumor necrosis factor α secretion (**B**) of mouse leukemic monocyte macrophages (RAW 264.7) were evaluated. The statistical difference (*p* < 0.05) among the samples at the same dose is indicated by different letters. Data are represented as mean ± standard deviation of four replicates.

The macrophage-stimulating activities of polysaccharides and polysaccharide-protein complexes from longan pulp have been confirmed in vitro and in vivo, including the upregulation of phagocytosis, nitric oxide (NO) production and cytokine secretion [4,5,7,8]. Compared with the control, LPPMs could significantly increase the NO production and tumor necrosis factor α (TNF-α) secretion of macrophages in the dose range of 100–400 μg/mL (*p* < 0.05), as seen in Figure 6. The initial activities of LPPMs were obviously weaker than those of 5 μg/mL lipopolysaccharide (LPS) (*p* < 0.05), except for the effect on NO production at 200 μg/mL. Dry-heating could effectively enhance the immunostimulating activity of LPPMs in the dose range (*p* < 0.05), and the enhanced level was associated with the treatment time. The NO production gradually decreased with an increase of dry-heating time. The TNF-α secretion increased to the maximum on the 2nd dry-heating day, and began to decrease on the 5th day.

The immunostimulating effects of polysaccharide and the polysaccharide–protein complex on macrophages are triggered via a specific receptor recognition, followed by intracellular signaling cascades leading to transcription activation and pro-inflammatory cytokine production [42]. Toll-like receptor (TLR) 4 and TLR2 are the main receptors responding to the action of polysaccharide from longan pulp [7]. It is suggested that the effect of dry-heating on the macrophage-stimulating activity of LPPMs may be related to the molecular structure-based TLR recognition. Generally, polysaccharides with a higher molecular weight exhibit stronger stimulatory effects, perhaps due to their highly repetitive structures which can cross-link receptors in a multivalent fashion [43]. The molecular weights of reported TLR4 polysaccharide-ligands widely range from 5 kDa to 2400 kDa, and this range is consistent with that of immunostimulatory polysaccharides [44]. Dry-heating induced change in molecular weight may be not the critical factor in improving immunostimulating activity, as LPPMs treated with and without dry-heating all show relatively low molecular weight. Polysaccharide–protein binding affinity is significantly weaker than protein–protein interaction [44], implying that the binding protein may enhance the interaction between the polysaccharide and the receptor for activating immune response. After dry-heating for 3 days, the polysaccharide–protein conjugates of LPPMs might undergo several degradation reactions [22], resulting in the decrease of immunostimulating activity.

## 3. Materials and Methods

### 3.1. Materials, Chemicals and Cells

Fresh ‘Shixia’ longan fruits were purchased from Zhongbai supermarket (Changgang Road, Wuhan, China). The fruits were manually peeled and seeded for use. The gel filtration calibration kit (6.5–75 kDa) was purchased from GE Healthcare (Buckinghamshire, UK). Mouse tumor necrosis factor-α (TNF-α) ELISA kit was purchased from Neobioscience Technology Co. (Shenzhen, China). Protein determination kit (Coomassie brilliant blue staining method) was obtained from Nanjing Jiancheng Bioengineering Institute (Nanjing, China). The cell counting kit (CCK-8) was purchased from EnoGene Biotech Co., Ltd. (Nanjin, China). The mouse RAW 264.7 macrophages, human hepatoma HepG2 cells and human gastric cancer SGC7901 cells were provided by Biosea Biotechnology Co., Ltd. (Wuhan, China).

### 3.2. Preparation of LPPMs

Polysaccharides and proteins were simultaneously isolated from longan pulp by an alkali-extraction and acid-precipitation method. Briefly, 1.0 kg pulps soaked in 1.5 L distilled water were homogenized at 10,000 r/min for 10 min. The homogenate was adjusted with 0.1 mol/L NaOH solutions to pH 8.0, agitated for 3 h on a magnetic stirrer at room temperature and then centrifuged at 4500 r/min for 10 min. After filtration through a Whatman No. 1 filter paper, the supernatant was adjusted with 0.1 mol/L HCl solutions to pH 5.0, followed by precipitation at 4 °C for 3 h. The precipitate was separated by centrifugation (4500 r/min, 10 min) and lyophilized to obtain LPPMs.

### 3.3. Dry-Heating Treatment of LPPMs

The uncovered weighing bottle (45 mm × 25 mm) containing 150 mg LPPMs was placed in the 60 °C oven equilibrated with saturated KBr solution for 1–6 days. After the dry-heating treatment, samples were lyophilized and kept in a desiccator at room temperature.

### 3.4. Composition Analysis

The content of polysaccharides was determined by the phenol-sulphuric acid method and expressed as glucose equivalents [45]; the content of uronic acids was determined by the method of Blumenkrantz and Asboe-Hansen, and expressed as glycuronic acid equivalents [46]; the content of proteins was determined using a protein determination kit according to its instruction; the level of free amino groups was determined by a modified o-phthalalehpyde (OPA) method and expressed as lysine equivalents [15].

### 3.5. UV-Visible Spectrophotometry

Samples were dissolved in distilled water. The UV-visible spectrum of 0.4 mg/mL sample solution was scanned in the wavelength range of 200–440 nm using a VU-1800 spectrophotometer (Shimadzu Corp., Kyoto, Japan). The absorbance of 4.0 mg/mL sample solution at 420 nm was measured.

### 3.6. FTIR Analysis

Samples were grounded with KBr powder, and then pressed into a 1-mm pellet for measurement in the frequency range of 4000–400 cm^−1^ by a Fourier transform infrared spectrophotometer (Nexus 5DXC FT-IR, Thermo Nicolet, Madison, WI, USA).

### 3.7. HPSEC Analysis

The HPSEC analysis was performed on a Waters Series System (Waters, Milford, MA, USA) mainly composed of a 2414 refractive index (RI) detector, a 2998 photodiode array (PDA) detector, a 1525 binary pump and an Ultrahydrogel 250 SEC column (7.8 mm × 300 mm). The detection wavelength of the PDA detector was 280 nm; 0.1 mol/mL sodium nitrate solution was used as the mobile phase at a flow rate of 0.5 mL/min; column temperature was 45 °C; the concentration of sample solution was 2.0 mg/mL, and 15 μL solution was injected after filtration through a 0.22 μm filter membrane. The standards with known molecular weight were used for calibration.

### 3.8. Antioxidant Activity Evaluation

DPPH radical scavenging activity was evaluated by the method of Brand-Williams et al. [47], with slight modifications. Briefly, a 1.5 mL sample solution was mixed with 0.5 mL of 0.2 mol/mL DPPH alcohol solution. After incubation under darkness at room temperature for 30 min, the absorbance of reaction solution at 517 nm was measured. The sample concentration resulting in 50% scavenging rate of DPPH radical (IC_50_ value, mg/mL) was calculated.

Hydroxyl radical scavenging activity was analyzed according to the method of Xiong et al. [48], with slight modifications. A 1-mLsample solution was added into a 5 mL centrifuge tube containing 1.0 mL FeSO_4_ solution (1.5 mmol/L) and 0.6 mL H_2_O_2_ (6.0 mmol/L). Then, 0.4 mL salicylic acid (2.0 mmol/L) was added in and shaken vigorously. After incubation in a 37 °C water bath for 1 h, the absorbance of reaction solution at 510 nm was measured. The sample concentration resulting in 25% scavenging rate of hydroxyl radical (IC_25_ value, mg/mL) was calculated.

Ferric reducing antioxidant power (FRAP) assay was performed with the method described by Jia et al. [49]. FRAP value (mmol/g) was expressed as millimoles of Fe^2+^ equivalents per 1 g of sample.

### 3.9. Antitumor Activity Evaluation

HepG2 cells or SGC7901 cells in the logarithmic phase were adjusted to 4 × 10^4^ cells/mL by Dulbecco’s modified Eagle medium (DMEM) containing 10% fetal bovine serum, and then plated in 96-well culture plates (100 μL/well) for 6 h incubation at 37 °C in a humidified 5% CO_2_ incubator (MCO-17 AIC, SANYO, Tokyo, Japan). After the removal of culture supernatant, a 200 μL/well medium containing a certain concentration of LPPMs (0, 50, 100, 200, 400 or 800 μg/mL) was then added in for 24 h incubation. The proliferation of tumor cells was detected by a CCK-8 kit according to its instruction. The sample concentration resulting in the 25% inhibition rate of tumor cells (IC_25_ value, mg/mL) was calculated.

### 3.10. Immunostimulating Activity Evaluation

The immunostimulating effects of the sample on the NO production and TNF-α secretion of macrophages were analyzed according to our previous method [7]. In brief, macrophage suspensions were added into a 24-well culture plate (2 × 10^8^ cells/well) and incubated for 3 h (37 °C, 5% CO_2_). The adherent cells were then incubated with 400 μL of medium containing 100–400 μg/mL LPPMs or 5 μg/mL LPS for 48 h. 200 μL medium was pretreated with ZnSO_4_ solution for the analysis of NO concentration by Griess method. The NO concentration was expressed as sodium nitrite equivalents (μmol/L). In addition, 200 μL medium were collected for the measurement of TNF-α concentration (pg/mL) using the mouse TNF-α ELISA kit.

### 3.11. Statistical Analysis

Data were expressed as means ± standard deviations. Significance of difference (*p* < 0.05) was evaluated with one-way analysis of variance followed by the Student–Newman–Keuls test using IBM SPSS Statistics 19 software (IBM, Armonk, NY, USA).

## 4. Conclusions

The homogeneous polysaccharide–protein mixtures (LPPMs) isolated from fresh longan pulp showed significant changes in physicochemical properties during dry-heating. The changes of the free amino group content, browning degree, infrared amide band intensity and molecular weight distribution, known as the characteristics of the polysaccharide–protein Maillard reaction, were indicated. The characteristics associated with the conjugation, rearrangement and degradation of the Maillard reaction reflected the different stages of reaction. However, the molecular mechanism of the reaction is still unclear, and the next-phase efforts on the elucidation of reaction products are necessary. Dry-heating-induced Maillard reactions could enhance the in vitro activities of LPPMs. At the advanced stage of the Maillard reaction, LPPMs showed relatively strong antitumor and immunostimulating activities, and those at the final stage possessed relatively strong antioxidant activities. The polysaccharide–protein-based Maillard reaction is suggested to be an effective method for improving the bioactivities of longan pulp polysaccharides, and the relationship between improving the effect and reaction stage needs to be deeply investigated.

## Figures and Tables

**Table 1:** Antioxidant activities of longan pulp polysaccharide-protein mixtures dry-heated for different times.

Dry-Heating Time (Day)	IC_50_ Value of DPPH Radical Scavenging (mg/mL)	IC_25_ Value of Hydroxyl Radical Scavenging (mg/mL)	FRAP Total Antioxidant Capacity (mmol/g)
0	1.398 ± 0.102 e	2.449 ± 0.092 d	0.038 ± 0.001 a
1	0.966 ± 0.012 d	1.095 ± 0.035 ab	0.066 ± 0.002 b
2	0.881 ± 0.014 c	1.015 ± 0.065 a	0.074 ± 0.002 c
3	0.551 ± 0.016 b	1.125 ± 0.075 ab	0.087 ± 0.001 d
4	0.556 ± 0.011 b	1.285 ± 0.055 c	0.107 ± 0.002 e
5	0.380 ± 0.020 a	1.215 ± 0.045 bc	0.119 ± 0.002 f
6	0.326 ± 0.008a	1.333 ± 0.024 c	0.146 ± 0.002 g

The statistical difference (*p* < 0.05) among the samples is indicated by different letters. Data are represented as mean ± standard deviation of three replicates.
